# 单细胞RNA测序在肺腺癌中的研究进展

**DOI:** 10.3779/j.issn.1009-3419.2021.102.19

**Published:** 2021-06-20

**Authors:** 逸楚 林, 东来 陈, 启峰 丁, 雪娟 朱, 蓉英 朱, 勇兵 陈

**Affiliations:** 1 215004 苏州，苏州大学附属第二医院胸心外科 Department of Thoracic Surgery, the Second Affiliated Hospital of Soochow University, Suzhou 215004, China; 2 200433 上海，同济大学附属上海市肺科医院胸外科 Department of Thoracic Surgery, Shanghai Pulmonary Hospital, Tongji University School of Medicine, Shanghai 200433, China

**Keywords:** 肺肿瘤, 单细胞测序, 耐药性, 演进, Lung neoplasms, Single-cell sequencing, Drug resistance, Progression

## Abstract

肺腺癌（lung adenocarcinoma, LUAD）是临床上肺癌最常见的亚型，是癌症相关死亡最主要的原因之一。过去十几年中，随着薄层计算机断层扫描（computed tomography, CT）广泛用于常规肺癌筛查，影像学上表现为小结节的LUAD发病率显著增高，其发生发展机制复杂，个体预后差异显著。尽管近年来针对LUAD的靶向和免疫疗法取得了重大进展，但肿瘤细胞的耐药性始终未得到有效解决，从而限制了患者获益。随着人类基因组计划的完成，以测序为基本手段的基因组学及转录组学进入临床和科研人员的视野。单细胞测序作为近年来受到高度关注的新型测序手段，与二代测序相比，其能在单细胞水平上对细胞群体进行特异性分析，揭示出每种细胞类型独特的变化，在单细胞水平上对许多异质基质细胞和癌细胞进行较精准地评估，从而揭示了分子成分的复杂性以及与非恶性组织中相应成分的区别。综上，通过单细胞测序深入了解LUAD发生发展机制和肿瘤微环境（tumor microenvironment, TME）的异质性及其耐药性形成机制，从而发现新的治疗靶点是临床医生和基础科学家迫切的需求。本文综合论述了单细胞测序在LUAD中的具体应用和研究进展。

## Abstract (en)

Lung adenocarcinoma (LUAD) is the most common subtype of lung cancer and one of the main causes of cancer-related deaths. In the past decade, with the widespread use of computed tomography (CT) in routine screening for lung cancer, the incidence of LUAD presenting as small pulmonary nodules radiologically, has increased remarkably. The mechanisms of the occurrence and progression of LUADs are complex, and the prognoses of patients with LUAD vary significantly. Although significant progress has been made in targeted therapy and immunotherapy for LUADs in recent years, the drug resistance of tumor cells has not been effectively overcome, which limits the benefits of patients. With the accomplishment of the Human Genome Project, sequencing-based genomic and transcriptomics have come into the field of clinical and scientific researches. Single-cell sequencing, as a new type of sequencing method that has captured increasing attention recently, can perform specific analysis of cell populations at single-cell level, which can reveal the unique changes of each cell type. Single-cell sequencing can also provide accurate assessment on heterogeneous stromal cells and cancer cells, which is helpful to reveal the complexity of molecular compositions and differences between non- and malignant tissues. To sum up, it is an urgent need for clinicians and basic scientists to deeply understand the pathogenesis and development of LUAD, the heterogeneity of tumor microenvironment (TME) and the mechanism of drug resistance formation through single-cell sequencing, so as to discover new therapeutic targets. In this paper, we reviewed and summarized the application and progress in single-cell sequencing of LUADs.

肺腺癌（lung adenocarcinoma, LUAD）是非小细胞肺癌最常见的一种组织学类型，在肺癌中的占比达到了50%^[1]^。2011年国际肺癌研究协会、美国胸科学会及欧洲呼吸学会提出了新的LUAD国际多学科分类，按镜下生长模式将LUAD主要分为鳞屑样生长、腺泡型生长、乳头型生长、微乳头型生长和实体型生长^[2]^。随着胸部薄层计算机断层扫描（computed tomography, CT）等筛查技术成为日常体检项目，早期LUAD检出率明显升高，在影像学上可表现为亚实性结节（纯磨玻璃结节和混合磨玻璃结节）和实性结节，其中亚实性结节多呈惰性生长，其患者预后往往优于实性结节^[3]^。目前对于LUAD的早筛早诊早治虽有所提高，但仍有近50%的术后患者会遭遇肿瘤复发转移^[4-6]^。此外，尚有少数LUAD患者在发现时病程已进入中晚期。随着靶向治疗和免疫治疗的开展，已有不少患者从中获益，但迄今为止个体的治疗敏感性差异和肿瘤的获得性耐药仍是LUAD多学科干预过程中最为棘手的难题。因此，探究LUAD发生发展的分子机制，明确LUAD对药物治疗的异质性来源，可避免早期肿瘤的过度治疗，有助于个体化精准治疗的开展。

单细胞测序是在单细胞水平上对细胞群体进行特异性分析的一项技术，其流程主要包括单细胞分离、细胞溶解与基因组DNA获取、全基因组扩增、测序与数据分析4个方面^[7, 8]^。流式细胞分选和激光捕获显微切割技术让单细胞的捕获成为可能^[8-11]^。目前主要采用基于条码（barcode）的单细胞识别来实现单细胞测序，即给每个细胞加上特定的DNA序列，测序时携带相同barcode的序列则视作来自同一个细胞^[7, 12]^。单细胞测序包括单细胞全基因组测序与单细胞转录组测序（single-cell RNA sequencing, scRNA-seq），可分别揭示基因组和转录组的变化，发现新的细胞亚型和基因，可用于存在高度异质性的干细胞及胚胎发育早期的细胞群体的基因调节网络的挖掘和亚细胞成分的基因表达谱的发现，与活细胞成像系统相结合，可在生理或病理情况下连续追踪基因表达的动力学变化，从而监测疾病的进展^[7-11]^。scRNA-seq可以用最小的样本开展无偏差高通量研究，测量基因表达水平更精确，在定量罕见变异和转录本时具有更高的灵敏度，其优势是全方位、多层次的。为使基础研究人员和临床医师从微观角度更好地加深对LUAD这种复杂疾病的认知，本文就近年来scRNA-seq在LUAD中的应用及研究进展作一综述。

## 1 scRNA-seq与LUAD的发生发展机制

### 1.1 早期LUAD的肿瘤微环境（tumor microenvironment, TME）特殊性

#### 1.1.1 早期LUAD与正常肺组织在单细胞水平上的差异

Lavin等^[13]^通过结合多元组织成像和配对的scRNA-seq分析比较28例Ⅰ期-Ⅲ期LUAD、非侵袭性肺组织（adjacent normal lung tissues, nLung）和血液的免疫特征，发现与nLung和血液相比，早期LUAD中肿瘤病变部位积聚了更多的免疫细胞，T细胞富集程度更高，与单核巨噬细胞浸润相关的CX3CL1表达水平略高，但是自然杀伤（natural killer, NK）细胞较少。质谱流式技术结果表明肿瘤浸润边缘三级淋巴样结构富集，在此结构中T淋巴细胞明显增多，巨噬细胞明显减少^[13]^。此外，不同表型T细胞在不同组织中分布不同，肿瘤部位的Treg细胞表达高水平的CTLA4、CD39、ICOS、41BB和低水平的CCR4，而细胞毒性CD8^+^ T细胞表达显著降低，非功能性T细胞富集，故T-效应因子/Treg比值降低，颗粒酶B和γ-干扰素（interferon γ, IFN-γ）的表达明显减少，NK细胞细胞毒性较低^[13]^。scRNA-seq分析鉴定出三个不同的表达高水平主要组织相容性复合体（major histocompatibility complex, MHC）II类分子的簇：CD1C、CCL22和CD207，推断为树突细胞（dentritic cell, DC）。与nLung和血液相比，肿瘤病变部位CD141^+^ DC（CD207）显著减少而CD1C^+^ DC（CD1C）略有增加，因此CD1C^+^ DC/CD141^+^ DC比值增加^[13]^。而早期LUAD中肿瘤浸润B细胞的IgG^high^亚群（浆细胞）具有通过产生免疫球蛋白抑制细胞生长的功能^[13, 14]^。这与Lambrechts等^[15]^和Wu等^[16]^的研究结论一致。故scRNA-seq可探究LUAD患者不同组织间各成分之间的差异，从而发现LUAD的发展变化及特征，有助于发现新的候选生物标志物与治疗靶点。

#### 1.1.2 鳞屑样生长的早期LUAD

既往研究已证实，含鳞屑样成分的LUAD预后优于不含有鳞屑样成分的LUAD，且鳞屑样成分在影像学上往往表现为亚实性、低密度的磨玻璃影^[17, 18]^，这种成分作为一种非浸润病变，恶性程度低、生长速度慢^[20]^。Lu等^[19]^对5例磨玻璃结节LUAD和5例实体LUAD进行scRNA-seq揭示两者肿瘤细胞及TME的异质性、各亚型组成分布：磨玻璃样LUAD的细胞增殖相关信号通路和内皮细胞中血管生成通路下调，成纤维细胞表达低水平胶原，T细胞免疫抑制途径激活，B细胞富集，呈递抗原，诱导激活NK细胞、肥大细胞从而进一步增强抑制肿瘤细胞增殖的免疫反应。此外，Xing等^[3]^针对上述问题对16例亚实性肺腺癌手术切除样本进行scRNA-seq，结合6例nLung和9例进展性肺腺癌（primary LUAD with lymph node metastasis, mLung）数据，揭示了亚实性肺腺癌TME的特征及发生发展特点，发现亚实性肺腺癌增殖相关途径与代谢途径上调，但处于nLung与mLung之间。而在免疫细胞方面，亚实性肺腺癌中效应CD8^+^ T细胞富集，缺乏耗竭CD8^+^ T细胞，杀伤耗竭比和CD4^+^ T细胞比例处于nLung与mLung之间，且CD16^+^ NK细胞亚群富集，呈现出明显的代谢紊乱和强烈的免疫监视杀伤应激状态^[3]^。这一结论反映了各细胞亚型在TME中发挥的作用并不相同，因此通过scRNA-seq阐明不同TME成分细胞特异性分子的表达水平以及基因与细胞表型的关系，为预测肿瘤的性质提供一定的参考。

#### 1.1.3 早期表皮生长因子受体（epidermal growth factor receptor, *EGFR*）突变LUAD

LUAD在年轻女性和从不吸烟的女性中发病率更高，值得注意的是，亚洲女性中LUAD中的*EGFR*突变普遍较高，可达40%-60%。有研究^[21]^对Ⅰ期/Ⅱ期*EGFR*突变LUAD样本和5个肿瘤邻近肺组织的共125, 674个细胞进行了scRNA-seq，发现早期LUAD样本中肺常驻巨噬细胞是最丰富的基质细胞群，高表达CD68但不表达CD11B，且白细胞介素1β（interleukin-1β, IL-1β）可诱导ELF3表达抑制，减弱2个LUAD细胞系A549（*KRAS*突变）和NCI-H1975（*EGFR*突变L858R）中核因子κB（nuclear factor kappa-B, NF-κB）通路基因的激活，引起细胞凋亡。研究人员^[21]^检测到一个同时表达效应T细胞及其功能障碍标志物的增殖亚型，该亚型内的T细胞主要表现出衰竭和调节性T细胞特征。上述研究提示scRNA-seq可用于鉴定*EGFR*突变LUAD的新增殖亚型，并为在部分靶向药干预患者中尝试免疫疗法提供了思路。

### 1.2 晚期LUAD

有研究^[22]^将转移性LUAD接受酪氨酸激酶抑制剂（tyrosine kinase inhibitor, TKI）靶向治疗前（TKI naive, TN）、治疗期间（residual disease state, RD）及表现出获得性耐药的疾病进展（progressive disease, PD）阶段的肿瘤样本进行scRNA-seq，发现TME可促进肿瘤细胞的异质性，且TME内成分基因突变丰富且复杂，鉴定了一个额外的新致癌突变——*EML4-ALK*致癌基因重排。在晚期LUAD的TME中载脂蛋白2和质膜微囊蛋白-1与肺癌生长迁移、进展和抗凋亡有关，TME中幼稚B细胞减少，IgG^high^ B细胞增多，可促进肿瘤细胞生长^[14, 23]^。转移性LUAD肿瘤上皮细胞和正常上皮细胞所共有的转录轨迹有明显调节差异，研究人员观察到一种有明确的肿瘤导向性（tumor-oriented）、侵袭性和异常增殖或凋亡特征的转录轨迹，这一转录轨迹的高表达与LUAD总生存率下降显著相关^[24]^。转移性LUAD中nLung的TME改变出现在病变早期，后期只是持续这种状态：①肿瘤上皮细胞具有高度血管生成和免疫功能低下的特性；②肌成纤维细胞逐渐取代了肿瘤基质中的基质成纤维细胞；③单核源性巨噬细胞和DC（CD163^+^ CD14^+^ DC）扩展并分化为总体抗炎表型；④B细胞被激活并在肿瘤组织中扩增；⑤细胞毒性NK细胞减少，调节性T细胞增加，衰竭T细胞表型晚期已经扩展到整个细胞毒性效应群体^[22, 24]^。基质和免疫种群的这些变化共同将具有免疫能力的组织转变为免疫抑制性TME。上述研究应用scRNA-seq比较LUAD不同治疗时间点TME的变化，对于了解LUAD发生发展机制、治疗方案的疗效及耐药机制提供了参考。

## 2 scRNA-seq与LUAD患者生存、预后及其标志物的发现

近期，一项研究通过对肿瘤细胞/正常上皮细胞和肿瘤T细胞/非肿瘤T细胞进行基于scRNA-seq的差异表达的配体-受体对分析^[28]^，鉴定出LUAD肿瘤细胞中65个配体-受体对和T细胞中96个配体-受体对。研究者选择其中11个可能显著影响生存结果的配体-受体对基因建立机器学习模型以准确预测LUAD患者的预后，并发现ITGB4、CXCR5和MET在预测模型中显著影响对预后判断的精准度^[28]^。此外，还有研究人员^[29]^利用scRNA-seq检测基因共表达网络，构建风险评分模型作为预测患者预后的工具，验证了风险评分模型riskScore是一个强大且独立的预后生物标志物，发现高风险Score亚型T细胞抑制性因子p53信号通路和调节性T细胞的激活，这可能是该亚型的预后较差的原因。肿瘤干细胞的研究对LUAD的早期诊断与预后有重大意义，但大多数研究主要在肿瘤干细胞上而非肿瘤干性。有研究^[30, 31]^通过scRNA-seq探究了肿瘤细胞干性指数（stemness index）及干性相关基因与患者预后的关系，发现与正常肺组织相比，LUAD中的mRNA干性指数更高，且晚期LUAD患者表现出更高的mRNAsi和较差的总体生存率；确定了8个差异表达的关键基因：*HSPA4*、*CDCA7*、*CDC20*、*CDK1*、*CLIP1*、*CCNB1*、*H2AFX*和*BLM*。其中*CDC20*、*CDK1*、*CCNB1*、*H2AFX*或*BLM*低表达的LUAD患者的总体生存率明显好转，而CLIP1低表达的总体生存率降低。此外，CDCA7的表达并不显著影响LUAD患者的总体生存率^[30]^。发现并鉴定了15个共表达的干性相关基因，其表达可能会影响肿瘤发生、进展，化学疗法和免疫疗法的疗效以及临床结果且其过度表达与LUAD中的免疫浸润减少有关，癌症干性与免疫力之间普遍存在负相关^[31]^。15个SRG可能在进一步验证后可用作潜在的候选生物标志物，作为预后指标和治疗靶标^[31, 32]^。通过免疫评分与基质评分，分析表达谱，确定5个基因（*CCR6*、*ITK*、*CCR4*、*DOK2*和*AMPD1*）为LUAD的预后因素。其中，*AMPD1*、*ITK*和*DOK2*的低表达与LUAD预后不良有关，CCR4是非小细胞肺癌总体生存率的独立危险因素，而肿瘤细胞中CCR6高表达是LUAD患者具有良好预后的独立预测因素^[32]^，此外高表达KIF2C的LUAD患者的总体生存率显著降低，“细胞周期信号传导途径”和“p53相关途径”显著富集，推测其可能通过调节细胞周期信号通路来预示不良预后^[33]^。因此，scRNA-seq可对差异表达的蛋白和基因等进行进一步分析与研究，发现新的基因特征、预后标志物以及相互作用关系。

## 3 scRNA-seq与LUAD患者治疗方案选择、优化及耐药机制的探索

### 3.1 IFN-γ信号通路表达下调——凡德他尼（Vandetanib）潜在的耐药机制

Vandetanib在*RET*重排的晚期非小细胞肺癌患者中有一定抗肿瘤作用，但疗效未得到肯定^[25]^，故Ma和他的团队^[26]^用scRNA-seq探究LUAD免疫应答相关基因的异质性，比较LC2/ad（Vandetanib敏感）与LC2/ad-R（Vandetanib耐受）细胞系，发现LC2/ad富集了较高水平的MHC Ⅱ基因与IFN-γ信号通路共表达基因，但LC2/ad-R内IFN-γ信号通路失协调表达且MHC Ⅱ基因表达水平较低，发现一种Vandetanib耐药的可能机制：IFN-γ信号通路失协调表达并表达较低水平的MHC Ⅱ基因，即MHC II和IFN-γ信号通路共同决定了免疫治疗耐药性的形成。

### 3.2 基因缺失介导的耐药机制

Maynard等^[22]^通过对PD肿瘤样本分析提出了靶向治疗的可能耐药机制，并证明肿瘤在治疗后异质性更为明显，检测到*KRAS*基因上新的突变（G13、G12C突变），提出肿瘤驱动基因缺失是一种可能的耐药机制；同时发现PD与TN、RD相比，参与Kynurenine通路的*IDO1*、*KYNU*和*QPRT*基因的表达显著增加，导致免疫抑制和免疫逃逸，可直接抑制免疫系统活性出现耐药。靶向治疗或免疫治疗后复发的LUAD可转变为肺鳞癌，原先对靶向药物敏感的LUAD出现耐药性正是由于腺癌转变为鳞癌。故Marynard等^[22]^获取并检测了一个具备*EGFR*外显子19缺失癌基因突变且使用EGFR抑制剂奥西替尼治疗的患者（TH226）在TN、RD与PD 3个治疗时间点的原发肿瘤部位样本，分析发现许多与鳞状细胞分化相关的基因（*KRT16*、*KRT14*、*KRT6A*、*KRT5*、*CLCA2*、*PKP1*、*ANXA8*、*DSG3*）在PD阶段比TN和RD阶段表达水平高，活检样本表明肿瘤由之前单纯的腺癌组织形态转变为鳞癌样生长，故致使药物疗效不佳，产生“耐药”。因此，scRNA-seq不仅能够监测癌症药物治疗过程中产生的生物分子和组织特性，还可以提供高分辨率的基因和通路水平视图，了解TME的变化及耐药机制，进一步指导治疗方案。

### 3.3 TME中与耐药形成有关的其他因子

刘亚飞等^[23]^对TME分析发现，在晚期LUAD单细胞转录组中胰岛素样生长因子结合蛋白7（insulin like growth factor binding protein 7, IGFBP7）表达下调能逆转耐药肿瘤细胞对EGFR-TKI的敏感性并促进肿瘤细胞凋亡，故推论出IGFBP7参与肿瘤细胞对EGFR-TKI耐药过程。除IGFBP7外，簇集蛋白、不均一核糖核蛋白A0和载脂蛋白2也与耐药性的形成有关。

免疫疗法在LUAD治疗中显示出不同的疗效，且常出现治疗不彻底现象，这可能是由于肿瘤细胞及其微环境的异质性所致。Guo等^[27]^对未接受过治疗的11例LUAD患者和3例肺鳞癌患者的12, 346个肿瘤细胞，邻近正常组织和外周血的T细胞进行scRNA-seq，发现不同组织中的T细胞组成与比例不同，肿瘤浸润淋巴细胞具有异质性，主要分为两大类：一类是“预衰竭”CD8^+^ T细胞、未激活的Tregs和激活的CD4^+^ T细胞；一类是衰竭T细胞和激活的Tregs；其中前者预后明显好于后者^[27]^。由此可知，通过scRNA-seq解读TME的异质性，对于“预衰竭”细胞的发现有利于患者分层，选择合适的免疫疗法。

## 4 讨论与展望

LUAD的肿瘤相关死亡率高，早诊早治是降低死亡率的重要手段，但是仅从放射影像学或组织病理学上进行精准分型难以实现，而scRNA-seq在分型方面具有更高的精准度，可在分子水平上对LUAD微环境和各类细胞进行检测。考虑到现有的肿瘤原发灶-淋巴结-转移（tumor-node metastasis, TNM）分期系统分期在评估个体预后方面仍存在较大局限性，基于TME的免疫评分作为一种用于定量病变区域免疫细胞浸润的预后指标，对补充TNM分期具有良好潜力^[34]^。既往多项研究^[3, 13, 21, 22]^表明LUAD有一种独立于TNM分期的预后相关免疫特征，利用scRNA-seq对TME中免疫细胞进行检测，有利于建立免疫分期和生存预测模型，改进TNM分期系统，进一步指导医疗决策。此外，有研究发现，在LUAD中微乳头和实性成分与更高的复发率相关^[35]^，而STAS阳性和淋巴结包膜外侵犯与更差的预后相关^[36]^，因此我们可以利用scRNA-seq对具有上述病理特征的LUAD进行TME解析，进而指导辅助治疗方案。

虽然scRNA-seq比RNA测序更精确，能检测到微量的基因表达子或罕见非编码RNA，技术有很大进展，但其覆盖率仍低，且细胞分离过程中可发生转录表达，大多数scRNA-seq数据集都有一定程度的污染。此外，细胞分离难易程度不一且有些细胞易被酶解和（或）机械破坏杀死，故产生的单细胞悬浮液很少能按比例代表许多起始细胞类型。最近发展的单分子测序技术无需逆转录和扩增步骤而直接对单个细胞的全长mRNAs进行测序，从而准确地检测基因不同亚型的表达水平，但高覆盖率、高保真性及高特异性的扩增及技术产生的误差仍然是亟待解决的问题。另外，各种数据分析软件（如Seurat、Sincera、NMF、ReF、RC、SCell等）对scRNA-seq的数据处理有重大意义，但对大量数据结果的专业分析仍是一个重大的挑战^[27]^。最后，目前高昂的价格亦限制了scRNA-seq的广泛应用。

综上所述，scRNA-seq有利于对各类LUAD的微环境解析及分子分型，建立免疫分期及预后模型，完善LUAD分期，制定最佳治疗方案。但scRNA-seq的广泛使用仍有很长的路要走，急需提高基因覆盖率和保真性，减少技术误差，发展技术手段和数据测试处理平台。
